# Circulating Levels of Osteopontin Predict Patients’ Outcome after Resection of Colorectal Liver Metastases

**DOI:** 10.3390/jcm7110390

**Published:** 2018-10-26

**Authors:** Sven H. Loosen, Daniel Heise, Cees H. Dejong, Sanchari Roy, Frank Tacke, Christian Trautwein, Christoph Roderburg, Tom Luedde, Ulf P. Neumann, Marcel Binnebösel

**Affiliations:** 1 Department of Medicine III, University Hospital RWTH Aachen, 52062 Aachen, Germany; sloosen@ukaachen.de (S.H.L.); sroy@ukaachen.de (S.R.); ftacke@ukaachen.de (F.T.); ctrautwein@ukaachen.de (C.T.); croderburg@ukaachen.de (C.R.); tluedde@ukaachen.de (T.L.); 2 Department of General, Visceral and Transplantation Surgery, University Hospital RWTH Aachen, 52062 Aachen, Germany; dheise@ukaachen.de (D.H.); chc.dejong@mumc.nl (C.H.D.); uneumann@ukaachen.de (U.P.N.); 3 Department of Surgery, Maastricht University Medical Centre, 6229 HX Maastricht, The Netherlands; 4 Division of Gastroenterology, Hepatology and Hepatobiliary Oncology, University Hospital RWTH Aachen, 52062 Aachen, Germany; 5 Department of General and Visceral Surgery, Klinikum Bielefeld, 33604 Bielefeld, Germany

**Keywords:** OPN, biomarker, CRLM, prognosis, CEA

## Abstract

For colorectal liver metastases (CRLM), surgical resection is the only potentially curative therapy, but even successfully resected patients often face disease recurrence, leading to 5-year survival rate below 50%. Despite available preoperative stratification strategies, it is not fully elucidated which patients actually benefit from CRLM resection. Here we evaluated osteopontin, a secreted glyco-phosphoprotein, as a biomarker in the context of CRLM resection. Tissue levels of osteopontin were analysed in CRLM using reverse transcription polymerase chain reaction (RT-PCR) and immunohistochemistry. Pre- and postoperative osteopontin serum concentrations were analysed by enzyme-linked immunosorbent assay (ELISA) in 125 patients undergoing resection of CRLM as well as 65 healthy controls. Correlating with an upregulation of osteopontin tissue expression in CRLM, osteopontin serum levels were significantly elevated in patients with CRLM compared to healthy controls. Importantly, high pre- and post-operative osteopontin serum levels were associated with a poor prognosis after tumour resection. Patients with initial osteopontin serum levels above our ideal cut-off value of 264.4 ng/mL showed a significantly impaired median overall survival of 304 days compared to 1394 days for patients with low osteopontin levels. Together, our data suggest a role of osteopontin as a prognostic biomarker in patients with resectable CRLM that might help to identify patients who particularly benefit from liver resection.

## 1. Introduction

Despite a decreasing incidence in Western countries, colorectal cancer (CRC) has remained one of the most common types of cancer worldwide, representing a major cause of cancer-related death [1]. The global incidence rates of CRC are approximately 35 and 50 cases per 100,000 population for women and men, respectively [2]. About 50% of CRC patients develop colorectal liver metastasis (CRLM) during the course of disease [3,4]. Hepatic resection has evolved as the standard curative intent therapy for CRLM patients in daily practice. Five-year survival rates after surgical resection of CRLM range from 25 to 58% [5,6], while CRLM patients who are enrolled in a palliative treatment are facing a dismal prognosis with a 5-year survival rate of less than 1% [6,7]. However, disease recurrence rates even after successful CRLM resection are high and about 65% of resected patients develop hepatic relapse within three years after surgery [4,8,9]. Importantly, the decision of interdisciplinary tumour boards whether or not to perform tumour resection in CRLM patients is often extremely challenging. Existing preoperative stratification algorithms such as the Fong score, which are mainly based on imaging techniques, the patients’ liver function and the clinical performance status, are often inconclusive and frequently do not sufficiently identify the subgroup of CRLM patients that actually benefit from surgical resection in terms of overall survival [10].

Osteopontin represents an extracellular glyco-phosphoprotein of 44 to 66 KDa, which plays a role in numerous biological processes such as cell migration, survival, angiogenesis, and inflammation [11,12]. Osteopontin has been associated with gastrointestinal malignancies such as hepatocellular carcinoma, cholangiocarcinoma and gastric cancer [13,14,15]. In primary CRC, tissue expression levels of osteopontin are upregulated and patients with high osteopontin levels show a reduced survival [16,17]. However, there are no sufficient data on the role of circulating osteopontin in the context of CRLM resection. Here, we evaluated the potential role of circulating osteopontin levels as a biomarker in a large cohort of 125 CRLM patients who underwent surgical tumour resection.

## 2. Materials and Methods

### 2.1. Study Design and Patient Characteristics

This retrospective observational cohort study was designed to evaluate circulating levels of osteopontin as a biomarker in patients undergoing liver resection for colorectal liver metastases (CRLM). Between 2011 and 2017 a total of *n* = 258 patients were admitted to the Department of General, Visceral and Transplantation Surgery at the University Hospital RWTH Aachen for surgical resection of CRLM. Out of these patients, *n* = 125 gave their informed consent to participate and were subsequently enrolled into this study (detailed patient characteristics are given in Table 1). Serum samples were collected prior to surgery and 6–7 days after tumour resection. The post-operative time point was chosen as many CRLM patients included in the study were referred to our centre from other peripheral clinics. Because some of these patients are transferred back to the initial clinic shortly after surgery, blood samples were collected at a rather early time point. The serum tubes were centrifuged for 10 min at 2000 g and the serum aliquots were stored at −80 °C until use. Moreover, we analysed 65 healthy, cancer-free blood donors. The study protocol was approved by the local ethics committee (University Hospital Aachen, RWTH University, Aachen, Germany) and conducted in accordance with the ethical standards laid down in the Declaration of Helsinki. Written informed consent was obtained from the patient or the appointed legal guardian.

### 2.2. Measurements of Serum Osteopontin Levels

Osteopontin concentrations were analysed using a commercial solid phase sandwich enzyme-linked immunosorbent assay (ELISA) according to the manufacturer’s instructions (Nr. 27158, Immuno-Biological Laboratories (IBL) Co., Ltd., Fujioka-Shi, Gunma, Japan). The ELISA uses Tetra Methyl Benzidine as a chromogen. The epitope of the used antibodies are as follows: (a) Coating antibody: anti-Human OPN (O-17) Rabbit IgG (IBL, Fujioka-Shi, Gunma, Japan); (b) Labeling antibody: anti-Human OPN (10A16) Mouse IgG MoAb Fab’-HRP (IBL, Fujioka-Shi, Gunma, Japan). The sensitivity of reaction is 3.33 ng/mL. The intra- and inter-assay variability at a measurement value of ~65 ng/mL shows a coefficient of variation (CV) of 4.7% and 7.8%, respectively. All samples were measured in duplicates. 

### 2.3. Semi-Quantitative Reverse Transcriptase PCR (qPCR)

Ribonucleic acid isolation from tissue samples, cDNA synthesis and semi-quantitative reverse transcriptase polymerase chain reaction (PCR) was performed as recently described according to the Minimum Information for Publication of Quantitative Real-Time PCR Experiments (MIQE) guidelines [15]. In detail, total RNA was purified from the tumour or healthy liver tissue using TRIzol reagent (Invitrogen, Carlsbad, CA, USA) and Directzol™ RNA MiniPrep (Zymo Research Europe GmbH, Freiburg, Germany) according to manufacturers’ instructions. Quantity and quality of the isolated RNA was measured using the Nanodrop (Thermo Scientific, Waltham, MA, USA). One μg of total RNA was then reverse transcribed using cDNA synthesis Kit H Plus (Peqlab, VWR International GmbH, Darmstadt, Germany). For reverse transcription, total RNA was reverse transcribed using miRNA miScript Reverse Transcriptase Kit (miRNA, Qiagen GmbH, Hilden, Germany) according to the manufacturer’s instructions. For qPCR, 2 μL of the cDNA samples were added in a total volume of 25 μL using SYBR Green ERTm (mRNA, Invitrogen) together with the specific primers or the miScript SYBR Green PCR Kit (miRNA, Qiagen) with miRNA-specific primers (Qiagen) and analysed using a standard PCR machine (Applied Biosystems 7300, Foster City, CA, USA). The following primers for SPP1 (osteopontin) were used: 1. For-SPP1: AGACCTGACATCCAGTACCCTG; 2. Rev-SPP1: GTGGGTTTCAG¬CACTCTGGT. All qPCR reactions were performed in duplicates. Data were generated and analysed using the SDS 2.3 and RQ manager 1.2 software packages.

### 2.4. Staining of Human CRLM Tumour Samples

For rehydration and antigen retrieval, 3-micron sections of paraffinised tissue were treated with PT-Link module (DAKO, Glostrup, Denmark) at pH6. Unspecific stain was blocked by hydrogen peroxide solution and Protein Block solution (both DAKO, Glostrup, Denmark). The primary antibody against osteopontin (polyclonal rabbit, Spring Bioscience, Pleasanton, CA, USA) was incubated to the sections at RT for 30 min, diluted at 1:100. Visualisation was performed by Envision Flex kit (DAKO, Glostrup, Denmark). After counterstain with hematoxylin, sections were dehydrated and coverslipped. Stained slides were scanned to obtain histological images (Hamamatsu, NanoZoomer 2.0 HT, Hamamatsu, Herrsching, Germany).

### 2.5. TCGA-COAD

The Cancer Genome Atlas Colon Adenocarcinoma (TCGA-COAD) database was accessed via the Oncomine Research Portal (https://www.oncomine.org). Osteopontin (SPP1) mRNA expression datasets were available for 101 CRC tumour samples as well as 19 healthy colon tissue samples as a control group. Box plot graphics were generated with the Oncomine visualisation online tool.

### 2.6. Statistical Analysis

Serum data are given as median and range. Non-parametric data were compared using the Mann–Whitney U Test and for multiple comparisons the Kruskal–Wallis Test (two-sided). Box plot graphics display a statistical summary of the median, quartiles and ranges. Receiver operating characteristic (ROC) curves were generated by plotting sensitivity against 1-specificity. The optimal cut-off values for ROC curves were established using the Youden Index (YI = sensitivity + specificity − 1). Kaplan–Meier curves were plotted to display the impact on survival. The optimal cut-off value for the identification of patients with an impaired overall survival (OS) was established by fitting Cox proportional hazard models to the dichotomised survival status (dead vs. alive) and the survival variable (survival time). The optimal cut-off is hereby defined as the point with the most significant (log-rank test) split. [18]. The prognostic value of variables was further tested by univariate and multivariate analysis in the Cox regression model. Inclusion criterion for multivariate testing was a *p*-value < 0.25 in univariate analysis. All statistical analyses were performed with SPSS 23 (SPSS, Chicago, IL, USA) [19]. A *p*-value of < 0.05 was considered statistically significant (* *p* < 0.05; ** *p* < 0.01; *** *p* < 0.001).

## 3. Results

### 3.1. Osteopontin Expression Is Upregulated in Colorectal Cancer and Colorectal Liver Metastases

**Figure 1:**
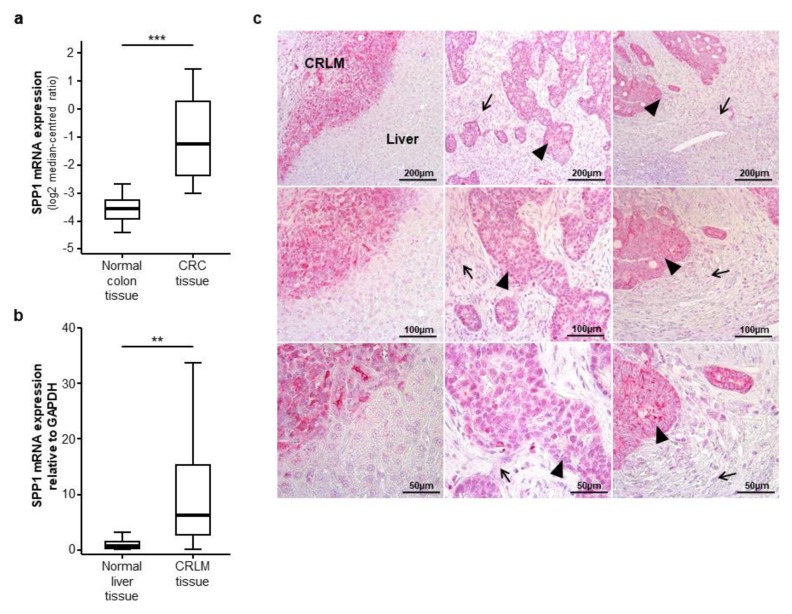
Osteopontin (SPP1) is overexpressed in colorectal liver metastases. (**a**) Osteopontin (SPP1) mRNA expression levels are strongly elevated in colorectal cancer (CRC) tumour samples compared to normal colon tissue samples (*u*-test, *p* (***) < 0.001); (**b**) SPP1 mRNA expression levels are significantly elevated in colorectal liver metastases (CRLM) tissue samples compared to normal liver tissue (*u*-test, *p* (**) < 0.01); (**c**) Immunohistochemical osteopontin staining in three exemplary CRLM samples (left, middle and right column). Left column: osteopontin is highly expressed in CRLM compared to normal liver tissue; middle and right column: tumour cells show a strong osteopontin expression (black arrow heads) while the peritumoral stromal tissue displays a weak osteopontin signal (black arrows); (upper panel: 100-fold magnification, middle panel: 200-fold magnification, lower panel: 400-fold magnification).

As a rational for evaluating circulating levels of osteopontin in CRLM patients, we first analysed osteopontin (SPP1) mRNA expression levels in primary CRC tumour samples. We therefore accessed the TCGA-COAD database and compared SPP1 mRNA expression levels in CRC tumour samples (*n* = 101) and healthy colon tissue samples (*n* = 19). Here, we found a highly significant overexpression of SPP1 in CRC tissue samples (Figure 1a). Subsequently, to evaluate a potential expression of osteopontin in CRLM, we analysed SPP1 mRNA expression levels in 20 CRLM tissue samples from our cohort using semi-quantitative reverse transcriptase PCR. Importantly, SPP1 expression was significantly upregulated in CRLM tissue compared to normal liver tissue (Figure 1b). In line with this, CRLM showed a strong immunohistochemical staining for osteopontin compared to the healthy liver tissue (Figure 1c, left column). Interestingly, while tumour cells display a strong osteopontin signal (Figure 1c, middle and right column, black arrow heads), we only observed a weak osteopontin expression in the surrounding stromal tissue (Figure 1c, middle and right column, black arrows).

### 3.2. Serum Levels of Osteopontin in Patients with Colorectal Liver Metastases

**Figure 2:**
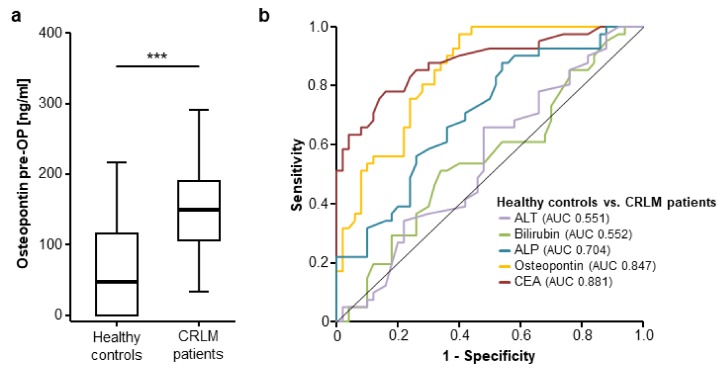
Circulating levels of osteopontin are elevated in patients with CRLM. (**a**) Circulating osteopontin levels are significantly elevated in patients with CRLM compared to healthy controls (*u*-test, *p* (***) < 0.001); (**b**) Osteopontin levels show a similar diagnostic power compared to carcinoembryonic antigen (CEA) serum levels in receiver operating characteristic (ROC) curve analysis, while serum levels of bilirubin, alkaline phosphatase (ALP) and alanine aminotransferase (ALT) are unsuitable for the differentiation between CRLM patients and healthy controls.

Following the important data on an overexpression of osteopontin in CRLM tissue, we subsequently analysed serum levels of osteopontin in patients with CRLM. Interestingly, osteopontin serum levels were significantly elevated in patients with CRLM compared to healthy control samples (Figure 2a, detailed serum levels are given in Supplementary Table S1). Receiver operating characteristic curve analysis revealed an area under the ROC curve (AUC) of 0.847 for osteopontin regarding the discrimination between patients with CRLM and healthy controls, which was only slightly inferior to carcinoembryonic antigen (CEA) (AUC_CEA_ 0.881, Figure 2b). Other standard laboratory markers for the assessment of liver function showed an inferior AUC for the discrimination between CRLM patients and healthy controls (AUC_ALT_ 0.551, AUC_Bilirubin_ 0.552, AUC_ALP_ 0.704). We established an ideal cut-off value of 130.25 ng/mL at which osteopontin showed a sensitivity and specificity of 63.2% and 87.7%, respectively, for the discrimination between CRLM patients and healthy controls (Figure 2b).

Subsequently, we investigated if the elevation of circulating osteopontin in CRLM patients is associated with specific disease characteristics (tumour grading, right- vs. left-sided primary CRC, KRAS mutated patients, patients with different ECOG PS). While we observed no difference in circulating osteopontin levels between patients with moderately (G2) or poorly (G3) differentiated tumours (Supplementary Figure S1a), left- or right-sided primary CRC (Supplementary Figure S1b), KRAS mutated and wildtype CRC patients (Supplementary Figure S1c), patients with normal or impaired ECOG performance status (PS) (Supplementary Figure S1d) as well as male and female patients (Supplementary Figure S1e), initial osteopontin serum levels were significantly elevated in patients with a high FONG score [10] (Supplementary Figure S1f).

### 3.3. Preoperative Osteopontin Concentrations are Associated with a Reduced Overall Survival after Resection of CRLM

**Figure 3:**
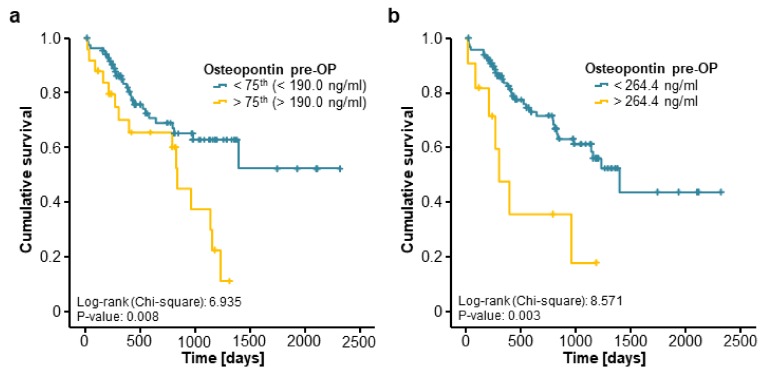
Elevated preoperative levels of circulating osteopontin are associated with a reduced overall survival after resection of colorectal liver metastases. (**a**) Patients with high preoperative osteopontin concentrations (>75th percentile) show a significantly impaired overall survival (OS) compared to patients with low osteopontin levels (log-rank test, *p* = 0.008); (**b**) Patients with initial osteopontin serum above our established ideal cut-off value (264.4 ng/mL) show a strikingly reduced median OS of 304 days compared to 1394 days for patients with osteopontin serum levels below this cut-off (log-rank test, *p* = 0.003).

We next evaluated if osteopontin serum levels might also have a prognostic value regarding the patients’ OS after tumour resection. Therefore, we divided our cohort of patients into two subgroups according to the preoperative osteopontin serum concentration (above or below the 75th percentile). Interestingly, Kaplan–Meier curve analysis revealed a significantly impaired OS for CRLM patients with initial osteopontin serum concentrations above this cut-off (Figure 3a). We next established an optimal prognostic osteopontin cut-off value of 264.4 ng/mL by fitting Cox proportional hazard models to the survival status and the survival time and testing for the most significant split in log-rank test [18]. When we applied this ideal cut-off value, the prognostic power of osteopontin serum levels was further increased, now showing a strongly impaired OS for patients with initial osteopontin serum levels above 264.4 ng/mL (Figure 3b). Importantly, the median OS of these patients was just 304 days compared to 1394 days for patients with initial osteopontin serum levels below the cut-off value. Additionally, long-term survival (beyond 5 years) was only reached by patients with initial osteopontin serum levels below 264.4 ng/mL.

We next performed extensive uni- and multivariate Cox regression analyses to further substantiate the potential prognostic function of osteopontin serum concentrations in this setting. In univariate testing, preoperative osteopontin serum levels above our ideal cut-off value were a significant prognostic factor for OS after resection of CRLM (HR 3.201 [1.405–7.292], *p* = 0.006). Moreover, in combined uni- and multivariate Cox regression analysis including markers of liver and kidney function (aspartate transaminase (AST) and creatinine), systemic inflammation (leucocyte and CRP), established CRC tumour markers (CEA) as well as clinical (ECOG PS, BMI) and pathological (largest size of CRLM, primary CRC tumour localisation) parameters, circulating levels of osteopontin stood out as an independent prognostic marker (HR 3.083 [1.054–9.020], *p* = 0.040, Table 2).

### 3.4. Postoperative Serum Levels of Osteopontin Are Associated with a Poor Prognosis after CRLM Resection

**Figure 4:**
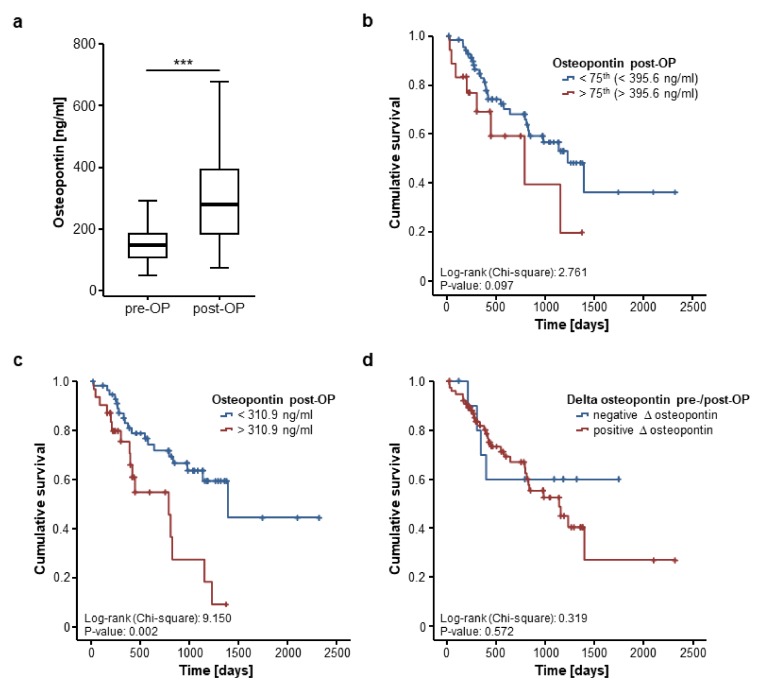
Postoperative osteopontin serum levels are associated with the patients’ prognosis. (**a**) Circulating osteopontin levels are significantly higher after tumour resection compared to preoperative levels (Wilcoxon signed-rank test, *p* (***) < 0.001); (**b**) Patients with postoperative osteopontin serum levels above the 75th percentile display a trend towards an impaired prognosis (log-rank test, *p* = 0.097); (**c**) At our ideal cut-off of 310.9 ng/mL, postoperative osteopontin concentrations significantly discriminate between patients with good or poor prognosis (log-rank test, *p* = 0.002); (**d**) Longitudinal changes of osteopontin serum concentrations before and after tumour resection are unsuitable to predict patients’ outcome (log-rank test, *p* = 0.572).

We further assessed if postoperative osteopontin serum levels are likewise associated with patient survival after CRLM resection. Postoperative serum samples were available for 103 patients and were significantly higher compared to the respective preoperative levels (Figure 4a). In line with our previous findings, postoperative osteopontin serum levels were unaltered between patients with moderately (G2) or poorly (G3) differentiated tumours (Supplementary Figure S2a), left- or right-sided primary CRC (Supplementary Figure S2b) and KRAS mutated or wildtype CRC (Supplementary Figure S2c). Moreover, osteopontin serum concentrations after CRLM resection did not differ between patients with normal or impaired ECOG PS (Supplementary Figure S2d) or male and female patients (Supplementary Figure S2e). However, we observed significantly elevated post-operative levels of circulating osteopontin in patients with a high FONG score [10] (Supplementary Figure S2f).

We subsequently divided our cohort of CRLM patients into two subgroups according to the post-operative osteopontin serum concentrations (above or below the 75th percentile). Using this cut-off value, patients with high post-operative osteopontin levels showed a strong trend towards an impaired overall survival but statistical significance was not reached (Figure 4b). However, when applying the ideal prognostic cut-off value that we again established as shown before, patients with postoperative osteopontin serum concentration above 310.9 ng/mL displayed a significantly impaired OS (Figure 4c). In line, univariate Cox regression analysis revealed postoperative osteopontin concentrations as a prognostic factor after CRLM resection (HR 1.002 [1.000–1.004], *p* = 0.046).

Finally, we evaluated if longitudinal changes before and after CRLM resection might have a prognostic value. Of the 103 patients with available post-operative serum samples, 91 patients showed increasing osteopontin levels after CRLM resection while 12 patients displayed decreasing serum levels. The mean delta of osteopontin before and after tumour resection was +161.33 ng/mL. We next compared the OS in patients with increasing or decreasing post-operative osteopontin serum concentrations. However, Kaplan–Meier curve analysis revealed no significant difference between these groups (Figure 4d).

## 4. Discussion

Recent progress in the interdisciplinary treatment of patients with advanced stage CRC has resulted in an improved overall survival even in patients with CRLM [20,21]. The implication of neoadjuvant chemotherapy and most importantly the extension of surgical indications by using novel techniques such as portal venous embolization and staged hepatectomies resulted in a markedly improved outcome for CRLM patients [4,21]. At the same time, locally ablative techniques (e.g., TACE, SIRT, and RFA) as well as systemic treatment options including highly active, personalised substances are gaining increasing attention even beyond clinical trials, resulting in prolonged survival even without tumour resection [22,23]. Consequently, for interdisciplinary tumour boards it often remains challenging to decide whether or not a patient should receive surgical resection of CRLM or rather be enrolled in a non-surgical treatment approach. Today, this decision is often based on imaging techniques, the patients’ liver function as well as the clinical performance status, while biomarkers that could provide information about the individual tumour biology are rarely taken into account [24,25]. Importantly, it was recently shown that not all potentially resectable CRLM patients actually receive surgical therapy [5]. Thus, novel biomarkers enabling the identification of patients who particularly benefit from CRLM resection in terms of overall survival might be a valuable addition to the existing stratification algorithms for patients with metastasised CRC.

In this study, we provided evidence that pre- and postoperative serum osteopontin concentrations represent a novel prognostic marker for patients undergoing resection of CRLM. We established an ideal prognostic cut-off value of 264.4 ng/mL for preoperative osteopontin serum levels above which CRLM patients showed a strikingly reduced median overall survival of only 304 days after tumour resection compared to 1394 days for patients with serum levels below this cut-off value. Importantly, only patients with initial osteopontin serum levels below this cut-off reached the 5-year survival mark. These results were further corroborated by uni- and multivariate Cox regression analysis including established tumour markers, markers of organ dysfunction as well as different clinicopathological parameters such as the localisation of the initial CRC or the largest diameter of CRLM as a surrogate for the tumour burden.

Osteopontin is a secreted extracellular protein, which has previously been associated with cancer and tumour progression [26,27]. Although the exact molecular mechanism by which high osteopontin levels negatively influence patient prognosis in our cohort of CRLM patients remains unclear, some potential pathophysiological explanations have recently been suggested. As such, osteopontin was shown to promote cell proliferation, migration and invasion and to inhibit cell apoptosis and autophagy by the p38 MAPK pathway [28]. Moreover, osteopontin expression in CRC cells and their cellular interactions with hepatocytes via integrin α-V and CD44v6 comprises an essential step in the process of CRC tumour spreading to the liver [29]. Finally, stroma-derived osteopontin has been associated with enhanced tumorigenesis through activation of the ERK signalling pathway resulting in an increased VEGF expression [30]. It is thus likely that the elevation of osteopontin serum levels observed in our cohort of CRLM patients is associated with high tumoral osteopontin expression that we confirmed for CRLM tissue samples. Moreover, the molecular pro-tumourigenic characteristics of osteopontin might be the origin for the impaired prognosis we observed in patients with high circulating levels of osteopontin. Interestingly, longitudinal changes of circulating osteopontin before and after surgery had no prognostic value on patient outcome. Thus, it is unlikely that postoperative osteopontin serum levels reflect a successful tumour resection in our cohort of patients. These findings suggest that higher circulating osteopontin levels were not exclusively evoked by the resected CRLM tumour cells but were also influenced by other cell types such as the peritumoral stroma and even more likely by non-tumorous liver remnants. To fully elucidate the longitudinal course of circulating osteopontin levels after surgery and their respective impact on the patients’ outcome, further analyses including several post-operative time points are needed. However, postoperative osteopontin serum levels in our cohort at day 6–7 were still able to discriminate between patients who showed long-term survival after tumour resection and patients who deceased early. It was recently shown that high osteopontin expression in the primary CRC tumour tissue represents a predictive factor for the occurrence of CRLM [31]. Moreover, the ratio of osteopontin expression in CRLM and the matched primary CRC samples also showed a prognostic significance [32]. In this context, it would be interesting to further evaluate whether circulating levels of osteopontin reflect this correlation and might therefore be used as a non-invasive biomarker for the prediction of CRLM or their recurrence after CRLM resection.

Importantly, this study has no predictive value regarding alternative treatment options for CRLM but only considered the prognosis of patients undergoing surgical tumour resection. Therefore, it is currently unknown if those patients stratified to have a poor post-operative outcome according to their individual osteopontin concentration, might have benefited to a greater extent from locally ablative techniques, systemic therapy or an active symptom control approach. In this line of thinking, it should also be considered that patients with a poor prognosis based on preoperative risk stratifications, e.g., including biomarker approaches, might especially benefit from a more aggressive therapy. As such, it was shown that BRAF mutant CRC patients, a subgroup of CRC patients who are usually facing a dismal prognosis, especially benefit from aggressive treatment regimens [33]. However, to further elaborate this approach and to confirm our findings on a prognostic role of osteopontin in patients undergoing resection of CRLM, larger prospective multi-centre studies are warranted in order to finally unravel the pivotal issue of identifying an optimal and personalised therapeutic approach for individual patients with advanced stage CRC. Our present study revealed osteopontin serum levels as a prognostic marker in patients undergoing CRLM resection and might therefore be a valuable addition to the existing preoperative stratification algorithms.

## Figures and Tables

**Table 1:** Characteristics of study population.

	Study Cohort
Patients with CRLM	125
Healthy controls	65
Sex (%): male–female	65.3–34.7
Age (years, median and range)	63 (5–85)
BMI (kg/m^2^, median and range)	25.5 (17.4–38.74)
Primary CRC characteristics (%):	
G2/G3	87.2/2.8
right-sided–left-sided	18.5/81.5
*KRAS* wt/*KRAS* mut	57.4/42.6
Clinical performance status (%): ECOG 0/1/2/3	66.4/32.0/0.8/0.8
Synchronous resection/metachronous resection (%)	13.6/86.4
Deceased during follow-up (%): Yes/No	37.3/62.7

**Table 2:** Uni- and multivariate Cox regression analyses for the prediction of overall survival.

	Univariate Cox Regression		Multivariate Cox Regression	
Parameter	*p*-Value	Hazard-Ratio (95% CI)	*p*-Value	Hazard-Ratio (95% CI)
OPN > 264.4 ng/ml	0.006	3.201 [1.405–7.292]	0.040	3.083 [1.054–9.020]
CEA	<0.001	1.001 [1.001–1.002]	0.018	1.001 [1.000–1.003]
Leukocyte count	0.014	1.168 [1.032–1.322]	0.146	1.121 [0.961–1.309]
CRP	0.003	1.017 [1.006–1.028]	0.910	0.999 [0.984–1.015]
AST	0.022	1.005 [1.001–1.009]	0.043	1.005 [1.000–1.011]
ALP	0.362	1.002 [0.998–1.005]		
Creatinine	0.978	1.023 [0.209–5.008]		
Sex	0.522	1.228 [0.655–2.302]		
BMI	0.332	1.033 [0.968–1.102]		
ECOG PS	0.165	1.534 [0.839–2.803]	0.436	1.344 [0.639–2.828]
Largest diameter of CRLM	0.096	1.089 [0.985–1.204]	0.380	0.946 [0.836–1.071]
Right- vs. left-sided primary CRC	0.064	1.910 [0.963–3.787]	0.051	2.320 [0.997–5.401]

OPN: osteopontin, CEA: carcinoembryonic antigen, CRP: C-reactive protein, AST: aspartate transaminase, ALP: alkaline phosphatase, BMI: Body-Mass-Index, ECOG PS: ECOG performance status, CRLM: colorectal liver metastasis, CRC: colorectal cancer.

## Supplementary Materials

The following are available online at http://www.mdpi.com/2077-0383/7/11/390/s1, Figure S1: Initial osteopontin serum levels and disease specific characteristics, Figure S2: Postoperative osteopontin serum levels and disease specific characteristics, Table S1: Serum levels of laboratory markers.
