# Supplementary material for: Circulating Levels of Osteopontin Predict Patients’ Outcome after Resection of Colorectal Liver Metastases
Source: J Clin Med. 2018 Oct 26;7(11):390. doi: 10.3390/jcm7110390 (PMC6262509; doi:10.3390/jcm7110390)
Supplement: Supplementary file 1 [file jcm-07-00390-s001.pdf]

Supplementary Materials:

Supplementary Table 1. Serum levels of laboratory markers.

	**CRLM patients** median [range]	**Healthy controls** median [range]
Osteopontin pre-OP [ng/ml]	149.2 [33.33-522.10]	46.7 [0-216.40]
Osteopontin post-OP [ng/ml]	280.5 [75.17-1255]	-
CEA [µg/l]	9.25 [0.30-1906]	1.25 [0.3-6.3]
WBC [cells/nl]	6.6 [1.9-18.5]	-
CRP [mg/l]	3.2 [0-120.6]	-
AST [U/l]	28.0 [2.1-399]	28 [20-78]
ALT [U/l]	23.5 [8-180]	20 [5-82]
GGT [U/l]	53 [10-1708]	17 [8-120]
ALP [U/l]	87.6 [41-479]	65 [36-102]
Bilirubin [mg/dl]	0.50 [0.10-1.29]	0.41 [0.1-1.46]
Creatinine [mg/dl]	0.84 [0.46-1.4]	-
Sodium [mmol/l]	140 [128-147]	-
Potassium [mmol/l]	4.4 [2.6-5.9]	-
Haemoglobin [g/l]	13.2 [8.2-16.9]	-
Platelets [cells/nl]	236.0 [102-782]	-

CEA: carcinoembryonic antigen, WBC: white blood cell count, CRP: C-reactive protein, AST: aspartate transaminase, ALT: alanine transaminase, GGT: γ-Glutamyl transpeptidase, ALP: alkaline phosphatase.

Supplementary Figure 1:

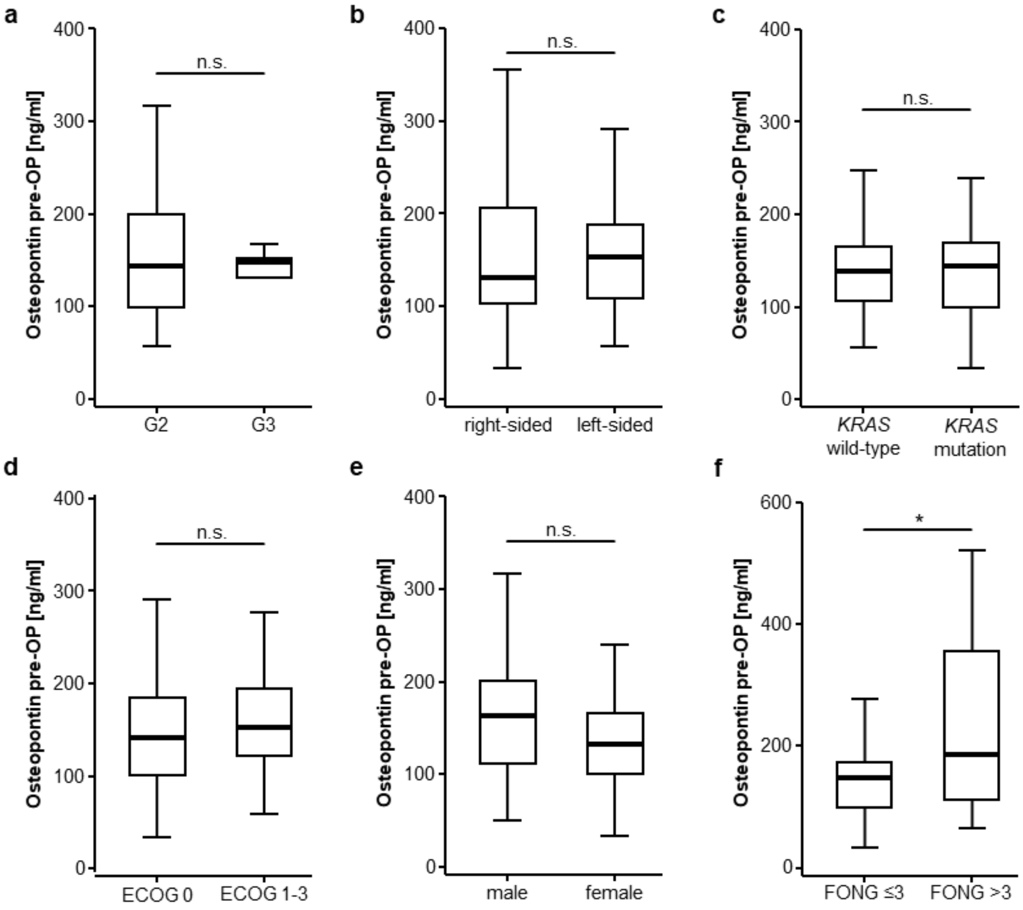

Supplementary Figure 1. Initial osteopontin serum levels and disease specific characteristics.

Initial osteopontin serum levels are unaltered between patients with moderately (G2) or poorly (G3) tumor differentiation (a, U-test, p = 0.639), left- or right-sided primary CRC (b, U-test, p = 0.337) or *KRAS* wildtype and mutated CRC patients (c, U-test, p = 0.977). There is no significant difference of osteopontin concentrations between patients with normal and impaired ECOG PS (d, U-test, p = 0.161) or male and female patients (e, U-test, p = 0.060). Osteopontin serum levels are significantly elevated in patients with a high FONG above 3 (f, U-test, p = 0.016).

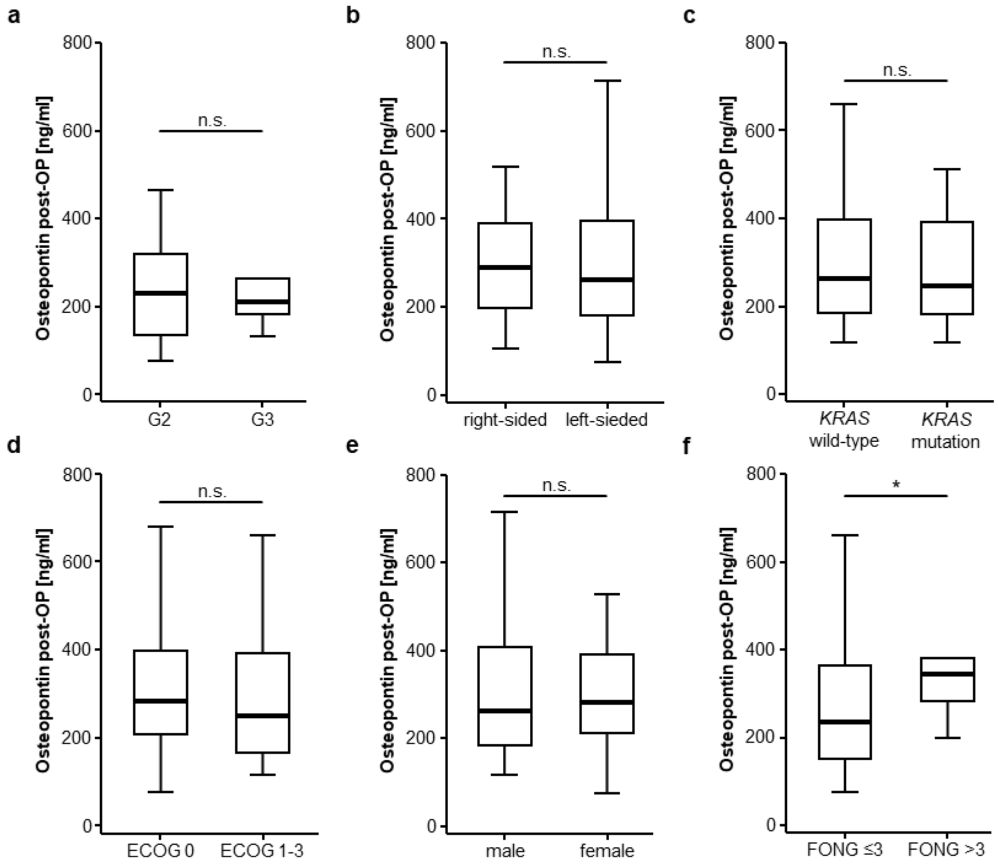

Supplementary Figure 2. Postoperative osteopontin serum levels and disease specific characteristics.

Postoperative osteopontin serum levels are unaltered between patients with moderately (G2) or poorly (G3) tumor differentiation (a, U-test, p = 0.983), left- or right-sided primary CRC (b, U-test, p = 0.986) or *KRAS* wildtype and mutated CRC patients (c, U-test, p = 0.893). There is no significant difference of osteopontin concentrations between patients with normal and impaired ECOG PS (d, U-test, p = 0.434), male and female patients (e, U-test, p = 0.966). Postoperative osteopontin serum levels are significantly elevated in patients with a high FONG above 3 (f, U-test, p = 0.045).
